# Halophytes play important role in phytoremediation of salt-affected soils in the bed of Urmia Lake, Iran

**DOI:** 10.1038/s41598-022-16266-4

**Published:** 2022-07-18

**Authors:** Fatemeh Ahmadi, Nayer Mohammadkhani, Moslem Servati

**Affiliations:** 1 Department of Soil Science, Faculty of Agriculture, Urmia University, Urmia, Iran; 2 Shahid Bakeri High Education Center of Miandoab, Urmia University, Urmia, Iran

**Keywords:** Plant ecology, Plant physiology, Plant stress responses

## Abstract

Soil salinity is a major threat in agriculture even in semi-arid regions of the world which can accelerate land degradation and desertification and decrease agricultural productivity and consequently jeopardize environmental and food security. Halophytes play important role in phytoremediation. This study is assessed the potential of *Halocnemum strobilace*ous, *Atriplex verruciferae*, *Salsola crassae*, and *Salicornia europaeae* in phytoremediation of saline soils occurred after water level desiccation of Urmia Lake. Three distances from the water body (500, 1000, and 1500 m) was selected for evaluating. Soils and plants were analyzed using standard methods. The mean values of salinity indices of the saline-sodic soil samples were identified as pH 8.6 and electrical conductivity (EC_e_) 65.34 dS m^−1^, also sodium adsorption ratio (SAR), and exchangeable sodium percentage (ESP) were higher than 13 and 15%, respectively. The maximum soil exchangeable Na^+^, K^+^, and Ca^2+^ concentrations (7200, 1900, and 1400 mg kg^−1^, respectively), also the concentrations of Mn^2+^ (12.5 mg kg^−1^), Fe^2+^ (5.5 mg kg^−1^), and Cu^2+^ (1.5 mg kg^−1^), were significantly different at various distances. However, the highest amounts were obtained at 500 m. In addition the concentration of Fe^2+^ (511.85 mg kg^−1^), Zn^2+^ (99.97 mg kg^−1^), and Na^+^ (25.65 mg kg^−1^) was the highest, especially in shoots. Furthermore, Salicornia and Halocnemum were more effective in salinity-remediation in comparison to other halophytes. The maximum dry matter (38%), protein (16%), and oil percentage (3.5%) were found in Salicornia, followed by Halocnemum. The findings indicated that salt-accumulating halophytes could be considered as the suggestions for phytoremediation saline soils and desalinating soil in arid and semi-arid regions.

## Introduction

Agricultural lands are affected by salinity all over the world, which has been estimated to become a worldwide problem in the upcoming decades^1^. More than 400 million hectares of agricultural lands are significantly influenced by salinity^2^. More than 6% of the world’s land has been recently recognized as salt-affected areas^3^, and 250 million hectares of agricultural lands have shown salinization with salt-saturated problems, which could be considered as approximately 50% of the total arable lands^4^. According to Kumar and Sharma^5^, salinity problems increased in 10% of arable lands annually. However, in Iran as an arid and semi-arid region, more than 23.8 million hectares of total agricultural lands and 3% of the irrigated lands are exposed to salinity problems^6^, which is more than 20% of the potentially irrigable agricultural lands in Iran^7^.

The Urmia Lake in northwest Iran has been recorded as the second most important saline lake in the world^7^. Decreased water level (more than 5 m) and increased salt concentration (185 to 220 g l^−1^) in this lake have recently caused serious salinity problems^8^ and could destroy the unique ecosystem of the lake. A recent study by Gholampour et al.^7^, found 50 cm thick salt deposits with more than 5000 km^2^ area.

Several studies have examined the effect of soil salinity on plant yields and growth parameters^8,9^ which could cause huge losses in plant productivity^10,11^. Salt accumulation in soils without suitable vegetation can increase wind erosion and cause the surface sediments to form a sleazy texture^12^. Transporting saline sediments with a high concentration of sodium chloride (NaCl) and other potentially fine-grain saline and toxic components could be dangerous for the ecological and environmental security, the establishment of vegetation, and human health in arid and semi-arid regions^9,13^.

Remediating salt-affected soils was established by various chemical and biological methods. However, using halophyte species as a natural, cost-effective, and useful phytoremediation method in saline soils has received growing research attention^14–16^ especially in situations with expensive and limited chemical amendments^17^.

Halophytes are classified as plants that can tolerate more than 1 M NaCl concentration in soils affected by salt^18^. Various strategies, ranging from inhibition to dramatic stimulation, have been recognized in halophytes for surviving under saline conditions^19^. Most halophytes can accumulate a high loading of soil ions in vacuoles with osmotic adjustment^20^. Previous studies showed that higher Ca^2+^/Na^+^ and K^+^/Na^+^ ratios in halophytes could increase the salinity tolerance^21,22^. Several studies evaluated the efficiency of using halophytes in improving saline and salt-affected soils^12,23^. Ventura et al.^24^, showed that the moderately saline (10 dS m^−1^) water could affect the flowering of neither *Salicornia* nor *Sarcocornia* species. In the same experiment, two *Sarcocornia fruticosa* genotypes were compared with the Salicornia species for biomass accumulation and a completely different cropping pattern. The results showed high tolerance of the species for salinity^25^. Singh et al.^26^, found that more than 69% of the examined saline lands were moderately suitable for cultivating *S. bigelovii*. Similarly, Song et al.^27^, reported the high tolerance of *Halostachys caspica, Kalidium foliatum,* and *Halocnemum strobilaceum* in saline conditions (0, 100, and 500 mM NaCl). Past studies indicated that *Atriplex nummularia* could show excellent adaptability to environments with high salinity and low water availability^28^.

Halophytes are efficient in adaptation and have a well-orchestrated mechanism for dealing with salinity stress^29^. In addition, they can complete their life cycle in saline conditions^30^.

Saline areas prone to the cultivation of these plants could be identified by determining the content of nutrients in halophyte plants in saline environments^31^. It is the hypothesis that halophytes can utilize the saline soils based on the plant species. So, the present study aimed to assess the relationship between soil nutrient content and nutrient absorption by some endemic halophyte plants (e.g., *Halocnemum strobilaceum*, *Atriplex verruciferae*, *Salsola crassae*, and *Salicornia europaeae*) which were grown in saline soils of north-western Iran. Using halophytes to stabilize the metals in the rhizosphere of saline and sodic soils prevents them from mobilization and migration in soil, groundwater, or air, and decreases their erosion, runoff, and leaching. Deciding how to assess the relationship between soil nutrient content and nutrient absorption by some endemic halophyte plants can be a challenge. We have tried to address this challenge in the present work and this is the main novelty of the work. The key objectives and novelties of this study are as follows briefly:

- To propose senior halophyte plant based on their efficiency of decrease soil salt in compare initial soil
- To carry out the halophyte plants edaphological and ecological assessment in salt- affected soils that evolved in the dried bed of Urmia Lake
- Comparing the Dry matter, protein, and oil percentage of each halophytes grown in different level of salt stress
- To investigate the effects of the different level of salt stress on the nutrient content of selected halophytes
- assess the relationship between soil nutrient content and nutrient absorption by some halophyte plants by calculating the transfer factor percentage

## Material and methods

### Study area and soil sampling

**Figure 1:**
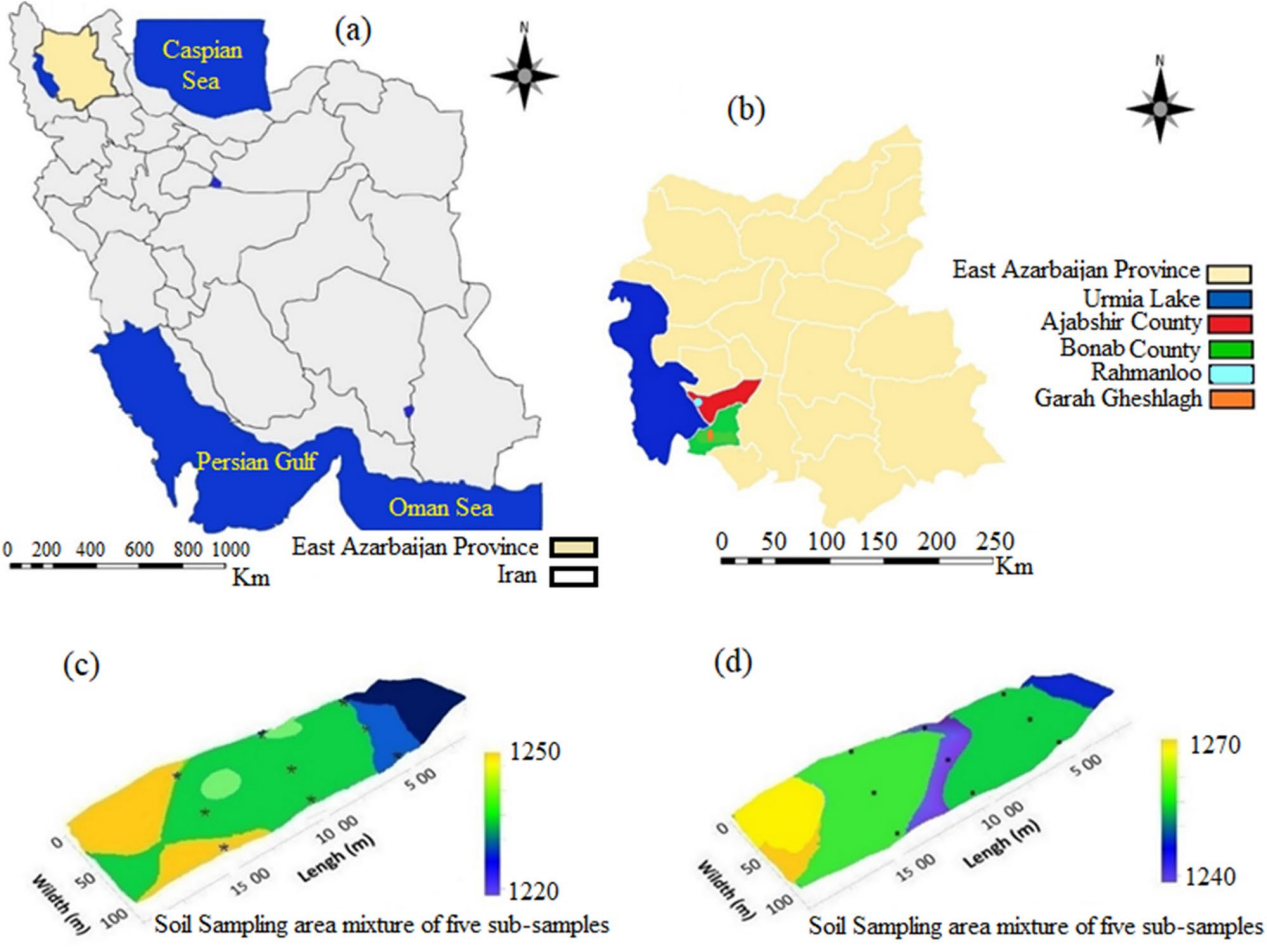
Location of (**a**) East Azarbaijan Province in Iran, (**b**) study area, (**c**) Rahmanloo sampling site, and (**d**) Gharagheshlagh sampling site. Figures were created in ArcGIS 10.2 (http://www.esri.com/software/arcgis).

Urmia Lake, the largest saltwater lake in the Middle East, is located between the provinces of East and West Azerbaijan, northwest Iran, with more than 85,000-hectare extension^7^. According to a recent study by Nhu et al.^32^, 8 billion cubic metric tons of salt have been accumulated in this lake. Two sites in the southeast of Urmia Lake, namely, Rahmanloo and Gharagheshlagh, were selected for the current study. As shown in Fig. 1, the two sites are located in Ajabshir (37° 30′ 22.3" N–45° 53′ 19.7" E) and Bonab (37° 13′ 53" N–45° 58′ 22" E) cities (Fig. 1).

In such low-slope studied areas with large lots of farmlands, the percolation of runoff into the aquifer through the infiltration can be expected. In terms of geology, 60% of the study area, especially in the central and east zones, has been covered by young alluvium, plain deposits, and fans^33^. A relatively narrow strip of salt clay zone has been stretched south and west of the study area. The north part of the plain has been covered by young terraces and alluvial deposits. A small part of the study area has been covered by other geological formations. There are some faults in the study area including normal fault, and probably, lineament, inferred, conceal fault^34^.

From 2001 to 2020, the mean annual temperatures of Rahmanloo and Gharagheshlagh were 16.0 and 15.4 °C, respectively, while their annual rainfalls were 345 and 350 mm, respectively. The temperature and moisture regimes in the regions are Thermic and Aridic (nearly Xeric), respectively. The soil parent material of the regions is the alluvial sediments of the Urmia Lake.

Soil samples were taken in the studied areas on 21 November 2020, along perpendicular transect on the Urmia Lake at three distances of 500, 1000, and 1500 m. At each distance, a sample consisting of five sub-samples was collected by a combined sampling method. Soil samples with different salinity levels were randomly collected at each site at the depth of 0–30 cm by using a stainless-steel auger and put into the polyethylene bags. The samples were air-dried at room temperature (25 ± 1 °C) and ground and sieved through 2-mm before chemical analysis.

### Chemical analysis of soil

The pH and electrical conductivity (EC) of soil were measured in soil saturated extracts by using a pH meter (Inolab pH 7110) and EC meter with a glass electrode (PAL-EC. Cat. No. 4331), respectively. Soil calcium carbonate (CaCO_3_) was measured after boiling 2.5 g of soil with 25 ml of 0.5 N HCl. Soil gypsum was determined using BaSO_4_ method. Soil cation exchange capacity (CEC) was determined after washing soil exchangeable sodium (Na^+^) ions with 1 M sodium acetate (NaOAc), 96% ethanol, and 1 M ammonium acetate (NH_4_OAc)^35^.

Soil Olsen phosphorus (Olsen-P) was measured based on the colorimetric method by spectrophotometer (model Cary 100) at 820 nm after extracting 1 g of air-dried soil sample with 20 ml of 0.5 M NaHCO_3_ (pH 8.5). Total nitrogen (N) was determined based on wet digestion according to Kjeldahl method^21^.

First of all, the easily soluble salts were washed using 96% ethanol to avoid the overestimation of exchangeable cations. Exchangeable potassium (K^+^) and Na^+^ were measured by extracting soil samples with 1 N NH_4_OAc by flame photometer^36^. Exchangeable magnesium (Mg^2+^) and calcium (Ca^2+^) concentrations were measured after extraction with EDTA using Atomic Absorption Spectrophotometer (AAS, Varian Spectra-220). The concentration of bioavailable heavy metals in soil (Cu, Fe, Mn, and Zn) was measured after extracting soil samples with the extractant consisting of 0.005 mmol l^−1^ DTPA (Diethylene triamine penta acetic acid), 0.01 mol l^−1^ CaCl_2_, and 0.1 mol l^-1^ TEA (tri ethanol amine)^37^. All analyses were performed at three replicates for controlling the accuracy of the results.

The sodium adsorption ratio (SAR), exchangeable sodium percentage (ESP), and total dissolved solids (TDS) are considered salinity indices^37^. The SAR is measured as the amount of sodium (Na^+^) relative to calcium (Ca^2+^) and magnesium (Mg^2+^) in the water extracted from a saturated soil paste^38^. The threshold values for SAR and ESP are 13 and 15%, respectively. The total dissolved solids (mmol l^-1^) are defined based on the EC of the soil saturated extract (EC_e_). These equations are respectively shown as follows^39^:

1 $${\text{SAR}} = \frac{{\left( {{\text{Na}}^{ + } } \right)}}{{\sqrt {({\text{Ca}}^{2 + } + {\text{Mg}}^{2 + } )/2} }}$$

2 $${\text{ESP}} = \frac{{{\text{Exchangeable}} \left( {{\text{Na}}^{ + } } \right)}}{{{\text{CEC}}}} \times 100$$

3 $${\text{TDS}} = 0.990 + 1.055 {\text{EC}}_{e}$$

### Plant analysis

**Figure 2:**
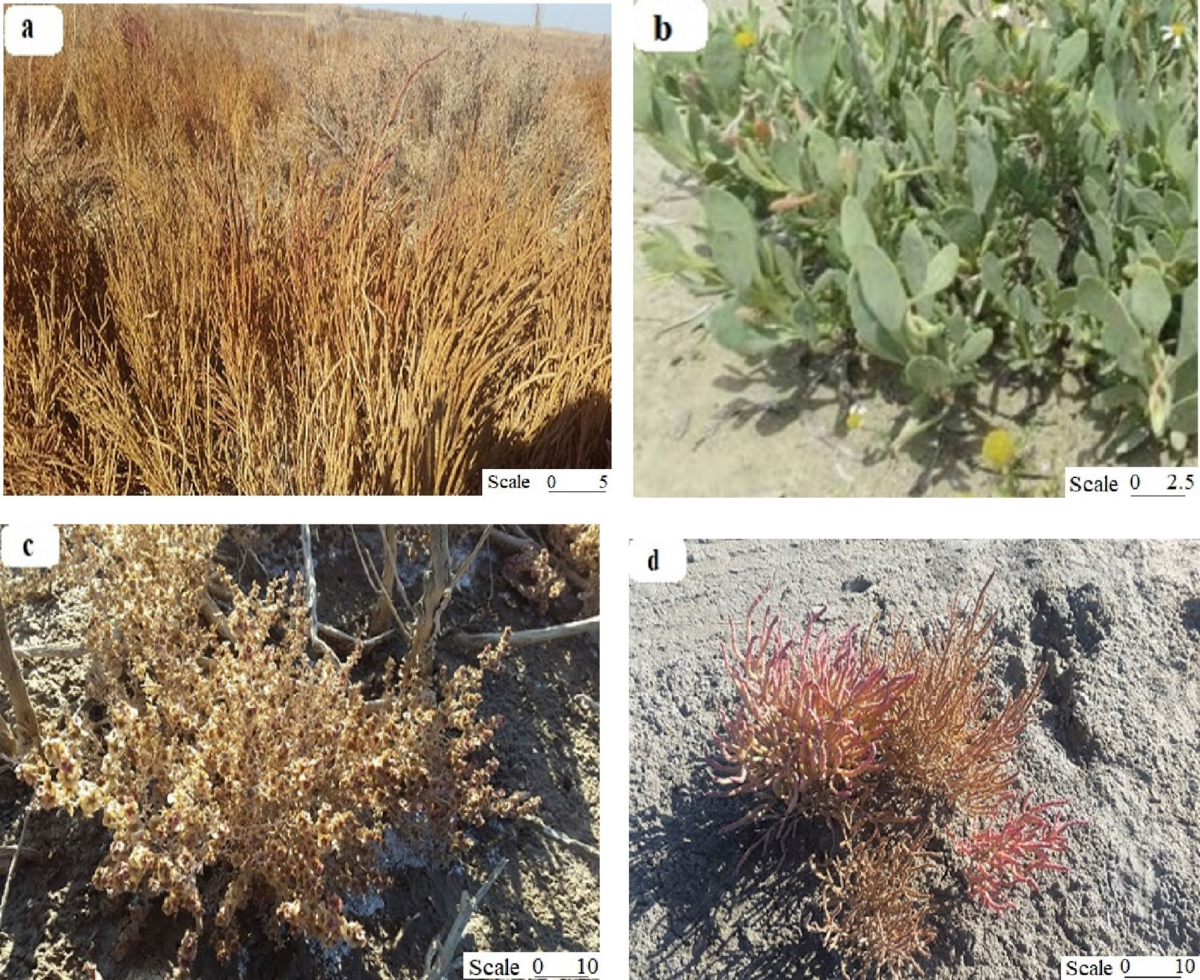
The studied plants: (**a**) *Halocnemum strobilaceum*, (**b**) *Atriplex verruciferae*, (**C**) *Salsola crassae*, and (**d**) *Salicornia europaeae* (Scale centimeter). Photographs were taken by M. Servati.

Different plants including *Halocnemum strobilaceum*, *Atriplex verruciferae*, *Salsola crassae*, and *Salicornia europaeae* were harvested (at the same age with same growth stage) at the end of the growing stage for eight months (21 November 2020) from Rahmanloo and Gharagheshlagh. The salinity threshold values for *Halocnemum strobilaceum*, *Atriplex verruciferae*, *Salsola crassae*, and *Salicornia europaeae* are 14, 9.4, 6.5, and 25 dS m^−1^, respectively^40^. The studied area also sampled for soil analysis (Fig. 2). The halophytes were collected according to the standard method in the plastic bags and separated into leaves, stems, and roots. The samples were oven-dried at 70 °C to a constant mass and estimated the soluble ions.

There are at least four principal plant communities in the Rahmanloo and Gharagheshlagh. Common species are *Halocnemum*, *Atriplex*, *Salsola*, and *Salicornia*. Grasslands are dominated by graminoids, that is, plant species belonging to Cyperaceae, Juncaceae, and Poaceae. The percentage cover of vascular species is about 25%, while the moss cover is 55%, and the average vascular plant leaf and moss biomass is about 35 and 370 g m^−2^, respectively.

The canopy was determined based on the standard method^41^. Various parts of the plants such as roots, leaves, and stems were separated and washed with tap water. Nine plants with comparable sizes which were not used for the experiment were selected for determining the biomass. Roots and shoot fresh weight were determined separately. To determine the amount of protein in a sample, 900 μl of distilled water was poured into 100 μl of the sample and 5 ml of Bradford reagent was added after mixing the contents of the tube. After 5 min, the light absorption of the sample was read at 420 nm and the standard protein of the unknown sample was obtained using the standard diagram and dilution coefficient of bovine serum albumin^5^. The oil percentage of the samples was determined according to previous research^42^. Soxhlet method was used for extracting 10 g of powder from shoot samples in n-hexane solvent for 24 h. After filtering with Whatman number one, the extract was concentrated in a rotary vacuum distillation machine at 41 °C. The oil percentage was obtained by dividing the oil weight by the sample weight. The percentage of dry matter of each plant was calculated based on the difference between their fresh and dry weights by standard methods^28^.

Phytochemical analysis was performed after drying the root and shoot samples at room temperature and powdered. Two grams of powdered samples were mixed with 25 ml solvent and shook for 180 min at 1000 rpm. The extracts were kept at 4 °C after filtration through Whatman filter paper (No. 1) (Whatman Ltd., England). Care was taken not to expose the extracts to light.

Dried and powdered samples were digested with the tri-acid mixture (H_2_SO_4_ + HNO_3_ + HClO_4_ in a 9:3:1 ratio) for photometrically determining K^+^ and Na^+^ in the extracts flame by using standard curves of K^+^ and Na^+^ for computation^12^. Chloride concentration was determined based on potentiometric titration. Calcium and Mg^2+^ concentration in the extracts was measured by potentiometric titration after mixing 0.5 g of samples with 40 ml of 0.5 N HCl^43^. The total concentration of Cu, Fe, Mn, and Zn was determined in the acidic filtrate using Atomic Adsorption Spectrophotometer^5^. Phosphorus (P) content was measured based on the vanadomolybdo-phosphoric acid yellow colour procedure using a Spectrophotometer at 410 nm after 30 min^44^. The nitrate (NO_3_^–^) concentration in the plant extracts was determined according to the standard method^45^. Briefly, 0.2 ml of the extract was mixed with 0.8 ml of 5% (w/v) salicylic acid in concentrated H_2_SO_4_. Nitrate concentration was determined using a spectrophotometer equipped with a rapid-sampling cuvette at 410 nm.

Transfer factor (TF) is an indicator that shows the accumulation of metals in plants in terms of their concentration in soil, it actually indicates the mobility of metals. The transfer factor can be calculated according to the following Eq. 4:

4 $${\text{Transfer factor}} \left( {{\text{TF}}} \right) = \frac{{\text{Metal concentration in plant tissue}}}{{ {\text{Metal concentration in soil}}}}$$

The metal concentration in plant and soil is based on mg kg^–1^ dry weight. The ratios higher than 1 show the accumulation of elements in plants while those lower than 1 indicate that the plants are not influenced by the elements. Plants with higher TF values could be used for phytoremediation^28^.

Duncan’s multiple range test was used for statistical analysis at the 0.05 probability level (*P* ≤ 0.05) using the Statistical Analysis Software (SAS, 9.4) program.

### Ethical approval

The authors declare that all relevant institutional, national, and international guidelines and legislation were respected.

## Results and discussion

### Comparison of soil salinity indices

Electrical conductivity in the saturation extract (EC_e_), ESP, and SAR have been suggested as the most important salinity indices in previous studies^5^, which is useful for soil management. Table 1 presents the critical ranges of these factors.

**Table 1:** Classification of salt-affected soils based on saturated paste extraction. *Source*: The Natural Resources Conservation Services (NRCS). ECe = EC of the saturation extract, SAR = Sodium adsorption ratio, ESP = Exchangeable sodium percentage.

Class	EC_e_ (dS m^−1^)	SAR	ESP (%)	pH	Soil structure
Normal	< 4.0	< 13	< 15	6–8	Flocculated
Saline	> 4.0	< 13	< 15	< 8.5	Flocculated
Sodic	< 4.0	> 13	> 15	> 8.5	Dispersed
Saline—Sodic	> 4.0	> 13	> 15	> 8.5	Flocculated

**Figure 3:**
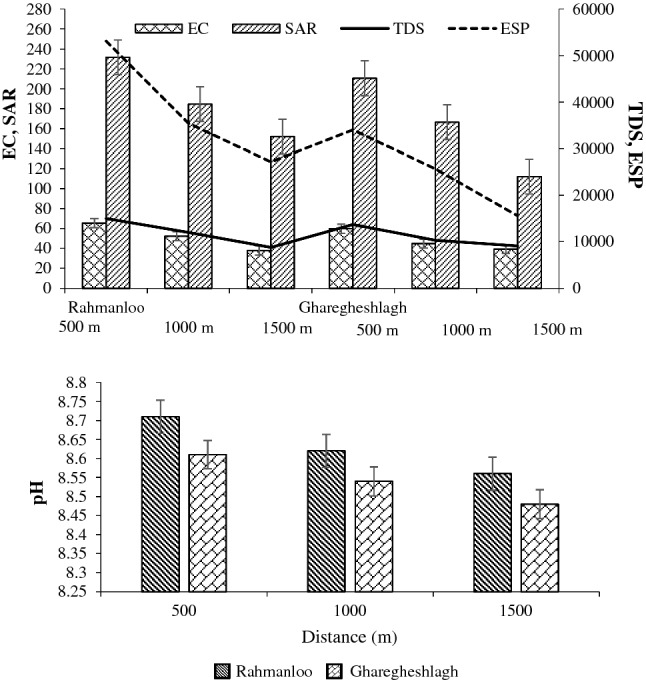
Soil salinity indices as affected by distance in two different areas.

All the studied soil samples had pH values higher than 8.5, which decreased by increasing the distance. The pH values in the Rahmanloo region were significantly higher than in Gharagheshlagh. The same trend was found for EC, except at the farthest distance (1500 m). Electrical conductivity in all soil samples not only was more than 4.0 ds m^−1^ but also 8.6 ds m^−1^ at the maximum level in the Rahmanloo studied area. The results showed that the SAR was 17 times more than the critical level, as shown in Table 1. The ESP of the samples was noticeably more than 15 in the two studied areas (Fig. 3). High values of ESP indicate an increase in sodicity in the soil exchange complex. Decreasing the ESP was obtained by increasing the distance, which is mainly related to a reduction of Na^+^ in soil exchangeable sited by increasing the distance and reduction of NaCO_3_ production with high solubility in soils that significantly affect the ESP^46^. Therefore, the studied soil samples could be classified as saline-sodic soils in both areas.

### Comparison of soil nutrients

**Figure 4:**
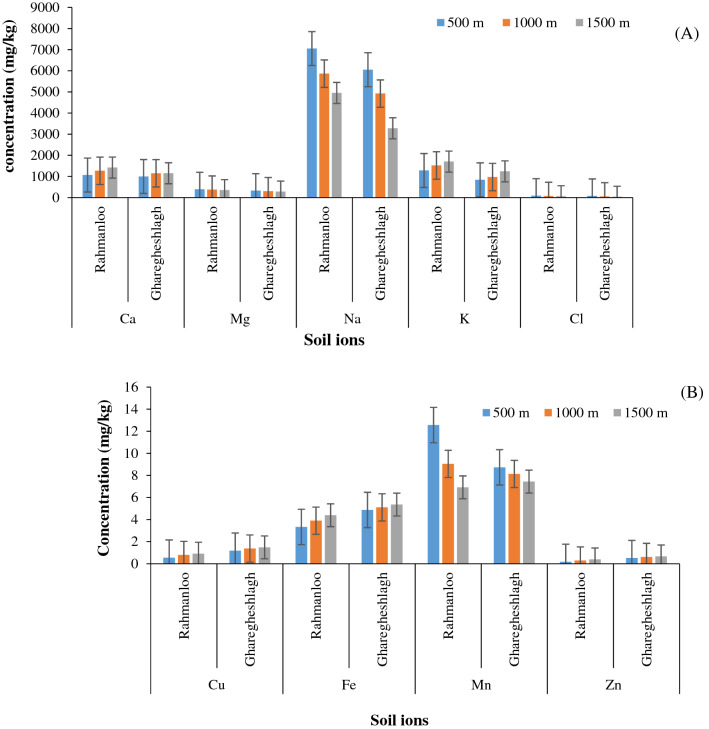
(**A**) Soil macro-nutrient and (**B**) micro-nutrient concentrations in the two studied areas.

Salt-affected soils can severely affect the availability of plant nutrients. Depletion in the fertility of such soils could be due to high levels of certain ions like sodium, carbonates, and bicarbonate overwhelming the accessibility of other ions like exchangeable calcium (Ca), potassium (K), phosphorous (P), iron (Fe), manganese (Mn), and zinc (Zn). The overall availability of phosphorus and micronutrients decreased due to the increase in soil pH (especially in sodic soils) during the reclamation process. Leaching salts was accompanied by leaching nutrients and decreased water uptake by the plants in the salt-affected soils ultimately led to decreased nutrient uptake due to physiological unavailability of the water. Therefore, the cations and anions concentration in the soil saturation extract was measured in this study. Figure 4 shows the results of soil chemical analysis.

As shown in Fig. 4, the exchangeable Na^+^, K^+^, and Ca^2+^ had the highest concentration in soil samples, followed by Mn^2+^, Fe^2+^, and Cu^2+^, respectively. As expected, the increase in pH and EC_e_ in saline-sodic soils increased the base cations. The solubility of micronutrients (e.g., Cu and Zn) was affected by pH, which decreased by increasing the pH^42^. However, previous researches Sherene^46^, Acosta et al.^47^ and Kadkhodaie et al.^48^, have shown that the solubility of some metals, especially Cu, in saline soils increases due to the formation of chloride complexes and a decrease in the surface charge density of soil colloids.

In the Rahmanloo region, soil available P increased from 4.18 mg kg^−1^ (on average) to 4.62 mg kg^−1^ at 500 m to 1500 m, respectively. The same trend was found in the Gharagheshlagh region. However, the concentration of soil P was higher in the Gharagheshlagh region (average 5.24 mg kg^−1^). In general, soil P concentration increased by increasing the distance. Previous studies showed the effect of soil carbonates on soil P concentration^49,50^. Accordingly, the calcium carbonate had a significant negative effect on P concentration. Complexation and precipitation of soil P carbonates could decrease the available P concentration^51^. The total content of soil N decreased by increasing the distance from the lake in both studied areas. The maximum total N was 0.081% and 0.122% in the Rahmanloo and Gharagheshlagh regions, respectively. Soil N content decreased by increasing the distance.

### Plant analysis

**Figure 5:**
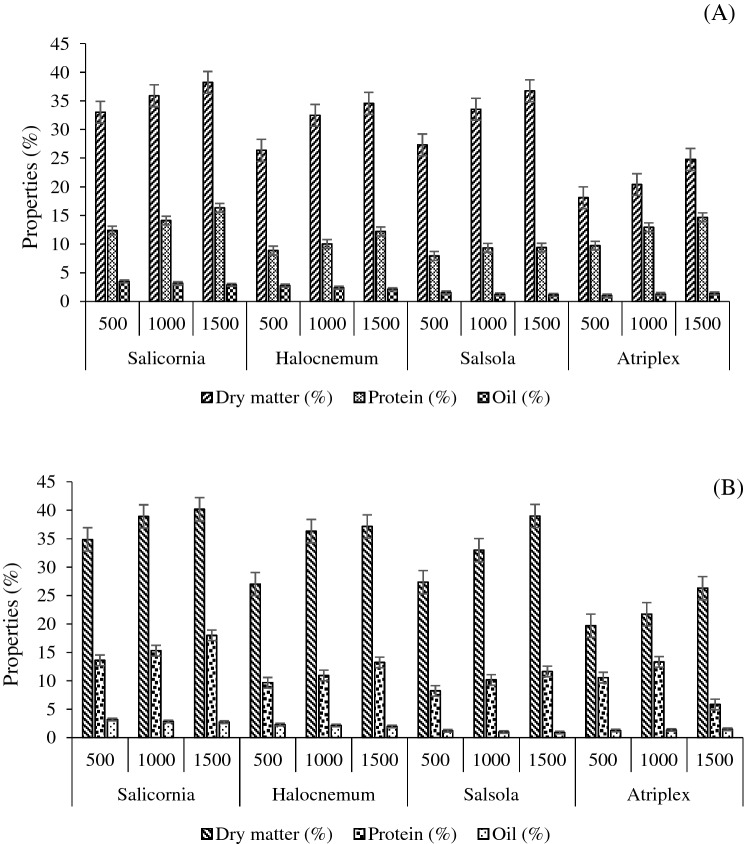
Dry matter, protein, and oil percentage of halophytes grown in (**A**) Rahmanloo and (**B**) Gharagheshlagh regions.

Together with a balanced nutritional composition, the presence of a wide variety of compounds such as protein and lipids with high nutritional value makes halophytes a valuable food source with functional properties^41^. Indeed, halophytes like Salicornia spp. have attracted attention in gourmet cuisine as an accompaniment or appetizer in fresh or elaborated salads^29^. The research of Martins-Noguerol et al.^38^, demonstrated the significant influence of salinity on the protein and lipids percentage of halophytes. So, it is important to pay attention to the influence of salinity on the bioactive compounds of halophytes. Figure 5 shows the dry matter, protein, and oil percentage of halophytes grown in the Rahmanloo and Gharagheshlagh regions. The results showed that the maximum dry matter (38.5%), protein (17.2%), and oil percentage (4.5%) were found in Salicornia than other halophytes (Fig. 5), which increased by increasing distance in all plants and could indicate the effect of high concentrations of basic cations in soil solution at shorter distances. In addition, higher percentages of dry matter, protein, and oil were found in the plants grown in the Rahmanloo in comparison to Gharagheshlagh (Fig. 5).

Tables 2 and 3 present the proximal compositions of the four halophytes. All macro and micronutrients are higher in shoot than in root, except for Ca^2+^, which may be related to the higher Na^+^ concentration in root tissue. Fe, Zn, Cu, Na, and Mn are the highest nutrients in plant tissues. The same results were found in the two studied areas. However, the plants cultivated in the Gharagheshlagh region had higher average nutrient concentrations in comparison to Rahmanloo (Tables 2 and 3). Based on the halophytes, significant differences were obtained. The results showed that *Salicornia* and *Halocnemum* could uptake more exchangeable nutrients than other plants. Different species have different responses in saline conditions. For example, plants can regulate the soluble products such as proline and glycine betaine for coping with salt stress and increasing the cell-volume^29^. Halophytes have developed distinct morphological, structural, and physiological strategies to survive in these high salt environments. Salt marsh halophytes cope with salt by excluding entry into roots, sequestering salts intracellularly (leading to succulence), and excreting salt via glands, usually on leaf surfaces^32^. Reduction of the Na^+^ influx, compartmentalization, and excretion of sodium ions are the main mechanisms of *Halocnemum* and *Salicornia* to cope with a high level of soil salinity^28^.

**Table 2:** The concentration of macro and micronutrients in different halophytes grown in Rahmanloo.

Plants	Root									Shoot								
	Cl (mg kg^−1^)	Na (mg kg^−1^)	K (mg kg^−1^)	Ca (mg kg^−1^)	Mg (mg kg^−1^)	Fe (mg kg^−1^)	Mn (mg kg^−1^)	Zn (mg kg^−1^)	Cu (mg kg^−1^)	Cl (mg kg^−1^)	Na (mg kg^−1^)	K (mg kg^−1^)	Ca (mg kg^−1^)	Mg (mg kg^−1^)	Fe (mg kg^−1^)	Mn (mg kg^−1^)	Zn (mg kg^−1^)	Cu (mg kg^−1^)
Salicornia	15.56	20.83	12.16	3.96	3.87	587.00	21.93	104.56	22.53	16.87	28.52	22.77	1.46	5.92	666.00	28.94	113.48	24.37
Halocnemum	15.3	19.16	8.63	3.55	3.77	488.55	16.31	97.95	20.42	16.20	25.71	17.70	1.31	5.83	580.55	24.27	105.04	21.54
Salsola	15.21	17.76	7.86	3.15	3.56	390.33	14.97	86.22	16.88	16.01	24.70	16.59	1.21	5.66	450.33	19.24	94.31	19.04
Atriplex	12.04	16.56	8.29	2.89	3.35	262.11	12.74	83.72	13.82	13.53	23.70	14.97	1.20	5.48	350.55	14.58	87.05	17.11

**Table 3:** The concentration of macro and micronutrients in different halophytes grown in Gharagheshlagh.

Plants	Root									Shoot								
	Cl (mg kg^−1^)	Na (mg kg^−1^)	K (mg kg^−1^)	Ca (mg kg^−1^)	Mg (mg kg^−1^)	Fe (mg kg^−1^)	Mn (mg kg^−1^)	Zn (mg kg^−1^)	Cu (mg kg^−1^)	Cl (mg kg^−1^)	Na (mg kg^−1^)	K (mg kg^−1^)	Ca (mg kg^−1^)	Mg (mg kg^−1^)	Fe (mg kg^−1^)	Mn (mg kg^−1^)	Zn (mg kg^−1^)	Cu (mg kg^−1^)
Salicornia	15.01	20.22	12.56	6.67	3.96	610.88	23.33	110.17	23.90	16.13	25.66	21.03	1.60	6.21	694.66	30.04	117.30	25.74
Halocnemum	13.17	17.90	11.17	6.53	3.75	509.11	18.22	100.64	20.57	14.77	24.00	17.97	1.51	5.94	634.44	26.24	112.28	23.27
Salsola	14.28	15.60	8.82	6.05	3.64	429.66	16.41	94.51	17.96	14.90	22.05	17.04	1.41	5.64	526.77	21.60	100.14	21.56
Atriplex	11.63	14.34	7.88	5.93	3.41	278.33	15.37	85.67	14.72	12.71	20.65	15.26	1.32	5.39	396.22	85.67	89.63	18.73

As the World Health Organization (WHO) recommends, it is necessary to have lower than 2 g sodium per day since higher amounts can be associated with cardiovascular diseases and blood pressure^52^. Nutrient contents in different halophyte species were varied although they were cultivated in the same saline conditions, which could be due to the plant mechanism for salting stress conditions. The obtained results in this study were higher than those reported by Gómez-Bellot et al.^30^, for Salicornia species. Based on the previous studies, the species of Salicornia and Halocnemum could accumulate the salts into epidermal vesicle bladders specialized in sodium sequestration, which could improve the tolerance of plants in salt-stress conditions^29^.

Halophytes are efficient in adaptation and have a well-orchestrated mechanism for dealing with salinity stress^19^. Halophytes can attain Na^+^ and Cl^−^ exclusion under high saline conditions, and Na^+^ exclusion from the xylem is performed through anatomical adaptations which reduce or prevent apoplastic movement of the solution from outside the roots to the xylem. Thus, cellular membranes and transporters can determine the ions which pass into the xylem^53^. To decrease the net uptake of salt ions to the shoots, halophytes display reduced stomatal opening, which can generate ROS. The capability of halophytes to handle ROS was studied in *the Eutrema parvulum*, which is a close relative of *Arabidopsis thaliana*^13^.

The reference values of Ca, K, Mn, Fe, Zn, and Cu were reported as 800, 2000, 375, 14, 10, and 1 mg, respectively. According to Mansouri et al.^54^, 2–10% of the aforementioned minerals can be supplied by consuming 20 g of fresh plants. The total concentrations of Ca and Mg in the studied plant species were different, which may be due to the pH of the cultivation media and the limitation of macronutrient absorption^28^.

Table 4 shows the amounts of nitrogen (N), phosphorus (P) concentration, and other soil properties in the studied areas. Soil N concentration decreased by increasing the distance in both areas, while a reverse result was found in soil P concentration. In addition, *Salicornia europaeae* had the highest N and P content in both areas. The plants grown in the Gharagheshlagh region had higher concentrations of N and P in comparison to the Rahmanloo region.

**Table 4:** The results of Nitrogen (N), Phosphorus (P) concentration, and other soil properties in the studied areas.

Site	Distance (m)	Soil N (%)	Plant N (%)				Soil P (mg/kg)	Plant P (%)			
			Salicornia	Halocnemum	Salsola	Atriplex		Salicornia	Halocnemum	Salsola	Atriplex
Rahmanloo	500	0.08	0.89	0.84	0.76	0.74	4.18	2.43	2.34	1.97	1.79
	1000	0.06					4.37				
	1500	0.07					4.62				
Gharagheshlagh	500	0.12	1.08	1.0	0.96	0.91	5.24	2.62	2.60	2.11	2.34
	1000	0.10					5.80				
	1500	0.10					5.20				

Site	Distance (m)	CaCO_3_ (%)	CaSO_4_ (%)	CEC (cmol_c_/kg)							
Rahmanloo	500	25.48	0.92	13.27							
	1000	26.10	1.07	16.54							
	1500	24.74	0.65	18.24							
Gharagheshlagh	500	19.54	0.82	17.78							
	1000	18.27	0.82	19.18							
	1500	17.26	1.1	21.07							

Different strategies are well recognized in halophytes for tolerating the salt conditions: antioxidant resistance, ion balance, and osmotic adjustment mechanisms. However, the mechanisms of plants are complex and not very clear^45^. Halophyte plants can maintain the water potential by osmotic adjustment, but various species may be different in the accumulated solutes and succulence^23^.

Feng et al.^9^, reported that osmotic adjustment can be controlled by biosynthesis of compatible solutes and accumulation/compartmentalization or exclusion of ions. Most plant species, especially halophytes, can maintain the water potential and necessary osmotic gradient for water uptake by accumulating different inorganic ions equal to or more than their concentration in soil solution^49^. The sequestration or accumulation of ions in vacuoles of halophyte plants can improve the water potential and protect the salt-sensitive enzymes in the cytoplasm^2^. The specific enzyme (salt-inducible Na^+^ /H antiporter) is necessary for exporting Na^+^ from the cytoplasm while the plant species can export the extra Na^+^ from their root cells or salt-excreting organs^55^. Meanwhile, the content of heavy metals was different in various organs of the plants.

**Figure 6:**
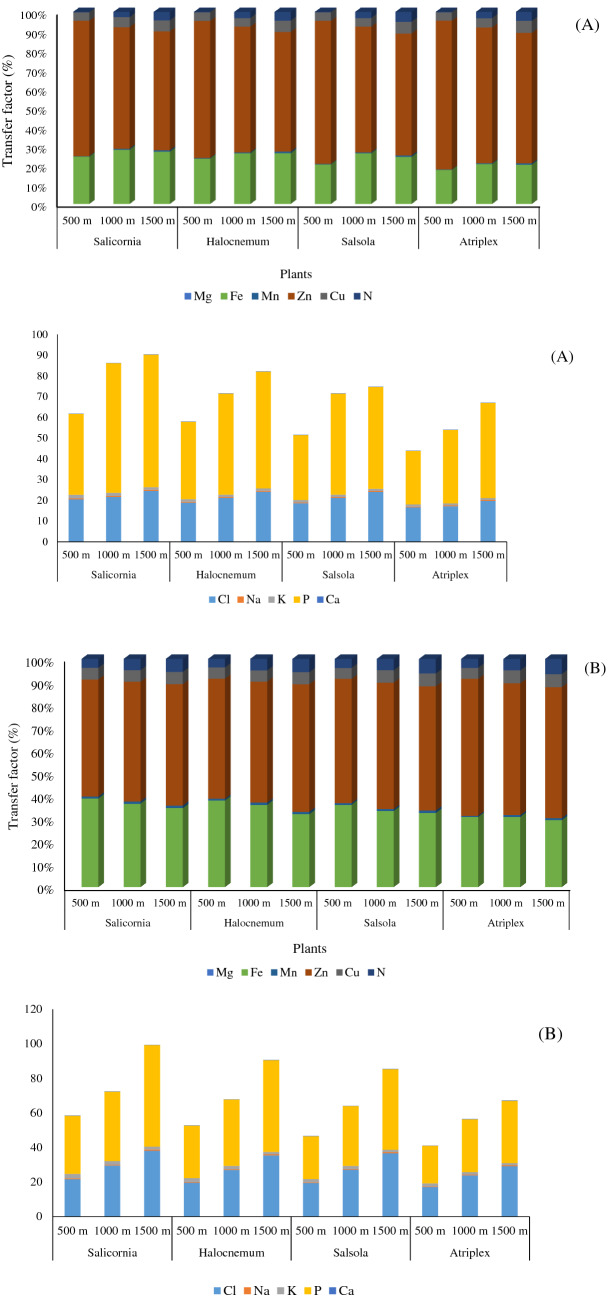
The transfer factor percentage in (**A**) Rahmanloo and (**B**) Gharagheshlagh regions.

Figure 6 shows the transfer factor of macro-and micro-nutrients. Zn, Fe, Cu, and N had the highest transfer factor percentage in both studied areas. However, the concentration of Zn in the plants grown in Gharagheshlagh was higher than in Rahmanloo, and the Fe concentration in the plants grown in the Rahmanloo was higher than in Gharagheshlagh (Fig. 6). The order of calculated TF is as Zn > Fe > Cu > Mn > N > P > Cl > K > Mg > Na (Fig. 6).

Zinc (Zn) values indicated that all the investigated species in this study had Zn accumulation capacity in their organs. The studied plants had a high Zn concentration. The TF values for Zn, Fe, Cu, and N in different vegetable crops considerably varied among the plant species, locations, and soil contamination. Different factors can influence the accumulation of nutrients in plants: soil cation exchange capacity, pH, moisture, element species, and climatic conditions ^41^. The continuous absorption of nutrients by plants during the growing period can increase the concentration of the nutrient, and even the soil has a lower concentration of nutrients.

Table 5 showed the correlation between salinity ions in plants and soil. There was a significant difference (*P* < 0.05, r > 0.7) between salinity ions (Cl^–^ and Na^+^) in roots and shoots of studied plants, also there was a significant difference between salinity ions in plant and soil.

**Table 5:** Correlation between salinity ions in plants and soil.

	Cl^−^ content of root	Cl^−^ content of shoot	Na^+^ content of root	Na^+^ content of shoot	Cl^−^ content of soil	Na^+^ content of soil
Cl^–^ content of root	1					
Cl^–^ content of shoot	0.955**	1				
Na^+^ content of root	0.737**	0.818**	1			
Na^+^ content of shoot	0.797**	0.853**	0.861**	1		
Cl^–^ content of soil	0.567**	0.594**	0.526**	0.731**	1	
Na^+^ content of soil	0.577**	0.598**	0.522**	0.736**	0.979**	1

^**^Showed a significant difference at *p* < 0.01.

**Figure 7:**
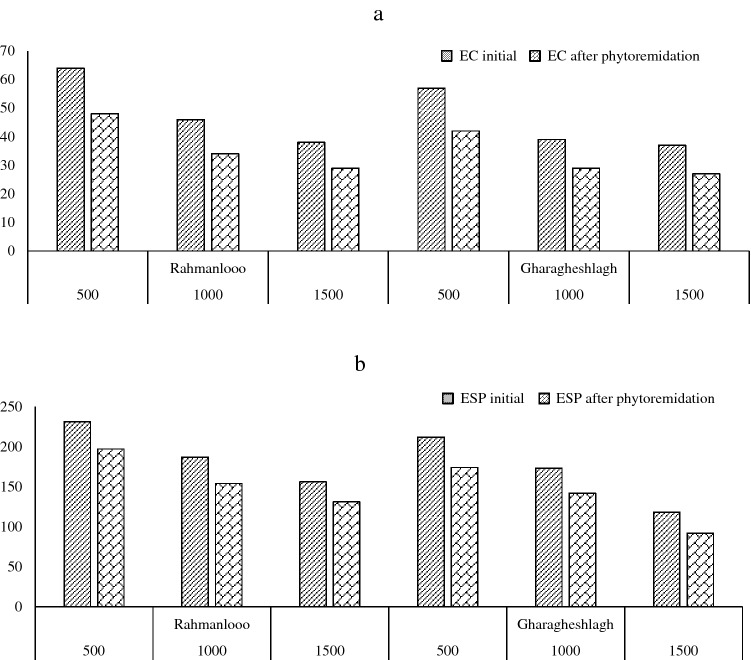
Comparison of EC (**a**) and ESP (**b**) in initial and after phytoremediation soil samples.

Figure 7 show that the growing of halophyte plants has been reduced the electrical conductivity (EC) and the exchangeable sodium percentage (ESP) of the soil, so these plants have high ability to salt phytoremediation. Therefore, halophytes with decrease salt (salt excluders and salt minimizer toxicity plants) from the saline-sodic soils can be increase the agricultural production yield and through this method, pressure on salt-tolerant plants would be decrease.

## Conclusion

The results showed differences between halophyte plants collected from the two saline-sodic studied areas. Soil samples were classified in saline-sodic soils with the maximum pH and EC values of 8.6 and 65.34 ds m^-1^, respectively. The SAR and ESP in all soil samples were higher than 13 and 15, respectively. The Mn^2+^, Fe^2+^, and Cu^2+^ had the highest concentration in soil samples, respectively. All macro and micronutrients, except for Ca^2+^, were higher in the shoot in comparison to root, and significant differences were obtained. The results showed that the *Salicornia* (67%) and *Halocnemum* (54%) could uptake more Mn^2+^, and Fe^2+^ compared to others. Based on the results, the best performance of the halophytes was found at high salinity levels. Thus, salt-accumulating halophytes are good suggestions for phytoremediation saline soils and desalinating soil in arid and semi-arid regions.

## Data Availability

The datasets used and/or analyzed during the current study are available from the corresponding author on reasonable request.

## References

[1] Lastiri-Hernández MA, Álvarez-Bernal D, Moncayo-Estrada R, Cruz-Cárdenas G, García JS (2020). Adoption of phytodesalination as a sustainable agricultural practice for improving the productivity of saline soils. Environ. Dev. Sustain..

[2] Plaut Z, Edelstein M, Ben-Hur M (2013). Overcoming salinity barriers to crop production using traditional methods. Crit. Rev. Plant Sci..

[3] Saghafi D, Delangiz N, Lajayer BA, Ghorbanpour M (2019). An overview of the improvement of crop productivity in saline soils by halotolerant and halophilic PGPRs. Biotech.

[4] Mirecki N, Agic R, Sunic L, Milenkovic L, Ilic ZS (2015). Transfer factor as an indicator of heavy metals content in plants. Fresenius Environ. Bull..

[5] Kumar P, Sharma PK (2020). Soil salinity and Food Security in India. Front. Sustain. Food Syst..

[6] Komaresofla BR, Alikhani HA, Etesami H, Khoshkholgh-Sima NA (2019). Improved growth and salinity tolerance of the halophyte Salicornia sp. by co-inoculation with endophytic and rhizosphere bacteria. Appl. Soil Ecol..

[7] Gholampour A, Nabizadeh R, Hassanvand MS, Taghipour H, Nazmara S, Amir Hossein Mahvi AH (2015). Characterization of saline dust emission resulted from Urmia Lake drying. J. J. Environ. Health Sci. Eng..

[8] Hafeez, M. B., Raza, A., Zahra, N., Shaukat, K., Akram, M. Z., Iqbal, S. & Basra, S. M. A. Gene regulation in halophytes in conferring salt tolerance. In Handbook of Bioremediation. Academic Press, 341–370 (2021).

[9] Feng L, Xia JB, Liu JT, Song AY, Chen YP, Zhao XM (2021). Effects of mosaic biological soil crusts on vascular plant establishment in a coastal saline land of the Yellow River Delta. China. Plant Ecol..

[10] Kearl J, McNary C, Lowman JS, Mei C, Aanderud ZT, Smith ST, Nielsen BL (2019). Salt-tolerant halophyte rhizosphere bacteria stimulate the growth of alfalfa in salty soil. Front. Microbiol..

[11] Yeo A (1998). Predicting the interaction between the effects of salinity and climate change on crop plants. Sci. Hortic..

[12] Arrekhi A, Gharmakher HN, Bachinger J, Bloch R, Hufnagel J (2021). Forage Quality of *Salsola turcomanica* (Litv) in Semi-Arid Region of Gomishan, Golestan Province. Iran. J. Rangel. Sci..

[13] Abuduwaili J, DongWei LIU, GuangYang WU (2010). Saline dust storms and their ecological impacts in arid regions. J. Arid land..

[14] Karakaş S, Cullu MA, Dikilitaş M (2017). Comparison of two halophyte species (*Salsola soda* and *Portulaca oleracea*) for salt removal potential under different soil salinity conditions. Turk. J. Agric. For..

[15] Manousaki E, Kalogerakis N (2011). A halophytes-an emerging trend in phytoremediation. Int. J. Phytoremediation..

[16] Mohebi Z, Khalasi Ahwaz L, Heshmati GA (2021). Comparison of Different Methods to Estimate Forage Production of Two Shrub Species *Halocnemum strobilaceum* (Pall.) Bieb and *Halostachys caspica* CA Mey (Case Study: Winter Rangelands of Golestan Province, Iran). J. Rangel. Sci..

[17] Öztürk, M., Altay, V. & Güvensen, A. Sustainable use of halophytic taxa as food and fodder: an important genetic resource in Southwest Asia. In *Ecophysiology, abiotic stress responses and utilization of halophytes*. Springer, Singapore. 235–257 (2019)

[18] Camacho-Sanchez M, Barcia-Piedras JM, Redondo-Gómez S, Camacho M (2020). Mediterranean seasonality and the halophyte *Arthrocnemum macrostachyum* determine the bacterial community in salt marsh soils in Southwest Spain. Appl. Soil Ecol..

[19] Jallali I, Zaouali Y, Missaoui I, Smeoui A, Abdelly C, Ksouri R (2014). Variability of antioxidant and antibacterial effects of essential oils and acetonic extracts of two edible halophytes: *Crithmum maritimum* L. and *Inula crithmoїdes* L.. Food Chem..

[20] Li B, Wang J, Yao L, Meng Y, Ma X, Si E, Wang H (2019). Halophyte *Halogeton glomeratus* is a promising candidate for the phytoremediation of heavy metal-contaminated saline soils. Plant Soil.

[21] Bradford MM (1976). A rapid and sensitive for the quantitation of microgram quantities of protein utilizing the principle of protein-dye binding. Anal. Biochem..

[22] Wang LM, Bu XL, Chen J, Huang DF, Luo T (2018). Effects of NaCl on plant growth, root ultrastructure, water content, and ion accumulation in a halophytic seashore beach plum (*Prunus maritima*). Pak. J. Bot..

[23] Holdt SL, Kraan S (2011). Bioactive compounds in seaweed: Functional food applications and legislation. J. Appl. Phycol..

[24] Ventura Y, Sagi M (2013). Halophyte crop cultivation: The case for Salicornia and Sarcocornia. Environ. Exp. Bot..

[25] Falasca SL, Ulberich A, Acevedo A (2014). Identification of Argentinian saline drylands suitable for growing Salicornia bigelovii for bioenergy. Int. J. Hydrog..

[26] Sherene T (2010). Mobility and transport of heavy metals in the polluted soil environment. BFIJ..

[27] Singh, D., Buhmann, A. K., Flowers, T. J., Seal, C. E. & Papenbrock, J. Salicornia as a crop plant in temperate regions: selection of genetically characterized ecotypes and optimization of their cultivation conditions. AoB plants 6 (2014). 10.1093/aobpla/plu071 PMC4268490 25387752

[28] Souza, E. R., dos Santos Freire, M. B. G., da Cunha, K. P. V., do Nascimento, C. W. A., Ruiz, H. A. & Lins, C. M. T. Biomass, anatomical changes, and osmotic potential in *Atriplex nummularia* Lindl. Cultivated in sodic saline soil under water stress. Environ. Exp. Bot. ENVIRON EXP BOT. **82**, 20–27 (2012).

[29] Delavar MA, Naderi A, Ghorbani Y, Mehrpouyan A, Bakhshi A (2020). Soil salinity mapping by remote sensing south of Urmia Lake. Iran. Geoderma Reg..

[30] Gómez-Bellot MJ, Lorente B, Ortuño MF, Medina S, Gil-Izquierdo Á, Bañón S, Sánchez-Blanco MJ (2021). Recycled wastewater and reverse osmosis brine use for halophytes irrigation: Differences in physiological nutritional and hormonal responses of *Crithmum maritimum* and *Atriplex halimus* Plants. Agronomy.

[31] Flowers TJ, Galal HK, Bromham L (2010). Evolution of halophytes: Multiple origins of salt tolerance in land plants. Funct. Plant Biol..

[32] Nhu VH, Mohammadi A, Shahabi H, Shirzadi A, Al-Ansari N, Ahmad BB, Chen W, Khodadadi M, Ahmadi M, Khosravi K, Jaafari A, Nguyen H (2020). Monitoring and assessment of water level fluctuations of the Lake Urmia and its environmental consequences using Multitemporal Landsat 7 ETM^+^ Images. Int. J. Environ. Res..

[33] Tavallaei S, Rashidi Ebrahim Hesari A, Fathi M, Farzaneh M, Mousavi S (2012). The evaluation of the geo-tourism for urban development: A case study in Ajabshir city, Iran. JCEU..

[34] Moghaddam MHR, Rouhi MN, Sarkar S, Rahimpour T (2018). Groundwater vulnerability assessment using the DRASTIC model under the GIS platform in the Ajabshir Plain, southeast coast of Urmia Lake, Iran. Arab. J. Geosci..

[35] Rowell, D. L. Soil science: Methods and applications. Harlow: Longman Group, p. 345 (1994).

[36] Thomas, G. W. Exchangeable cations. Methods of soil analysis: Part 2 chemical and microbiological properties, **9**, 159–165 (1983).

[37] Chaudhary, D. Ion accumulation pattern of halophytes. In Halophytes and climate change: adaptive mechanisms and potential uses. CAB International. 137–151 (2019).

[38] Martins-Noguerol R, Cambrollé J, Mancilla-Leytón JM, Puerto-Marchena A, Muñoz-Vallés S, Millán-Linares MDC, Moreno-Pérez AJ (2021). Influence of soil salinity on the protein and fatty acid composition of the edible halophyte Halimione portulacoides. Food Chem..

[39] Riasi A, Mesgaran MD, Stern MD, Moreno MR (2008). Chemical composition, in situ ruminal degradability, and post-ruminal disappearance of dry matter and crude protein from the halophytic plants *Kochia scoparia*, *Atriplex dimorphostegia*, *Suaeda arcuata*, and *Gamanthus gamacarpus*. Anim. Feed Sci. Technol..

[40] Kafi, M. & Salehi, M. Potentially domesticable chenopodiaceae halophytes of Iran. In Sabkha ecosystems. Springer, Cham. 269–288. (2019).

[41] He HB, Li Y (2008). Study on measures of biomass allocation of two desert halophyte species under drought and salt stress. Arid Zone Res..

[42] Lei W, Zhen-Yong Z, Ke Z, Chang-Yan T (2012). Oil content and fatty acid composition of dimorphic seeds of desert halophyte *Suaeda aralocaspica*. Afr. J. Agric. Res..

[43] Cottenie, A. Soil and plant testing as a basis of fertilizer recommendations (No. 38/2). (1980).

[44] Estefan, G., Sommer, R. & Ryan, J. Methods of soil, plant, and water analysis. A manual for the West Asia and North Africa region, **3**, 65–119 (2013).

[45] Manchanda HR, Sharma SK, Singh JP (1985). Effect of increasing levels of residual sodium carbonate in irrigation water on the exchangeable sodium percentage of sandy loam soil and crop yield. J. Indian Soc. Soil Sci..

[46] Song J, Feng G, Zhang F (2006). Salinity and temperature effects on germination for three salt-resistant halophytes, *Halostachys caspica*, *Kalidium foliatum*, and *Halocnemum strobilaceum*. Plant Soil.

[47] Acosta JA, Jansen B, Kalbitz K, Faz A, Martínez-Martínez S (2011). Salinity increases mobility of heavy metals in soils. Chemosphere.

[48] Kadkhodaie, A., Kelich, S. & Baghbani, A. Effects of salinity levels on heavy metals (Cd, Pb, and Ni) adsorption by sunflower and sudangrass plants. B.E.P.L.S. **1**(12), 47–53 (2012).

[49] Meyer G, Frossard E, Mäder P, Nanzer S, Randall DG, Udert KM, Oberson A (2018). Water-soluble phosphate fertilizers for crops grown in calcareous soils–an outdated paradigm for recycled phosphorus fertilizers?. Plant Soil.

[50] Teng Z, Zhu J, Shao W, Zhang K, Li M, Whelan MJ (2020). Increasing plant availability of legacy phosphorus in calcareous soils using some phosphorus activators. J. Environ. Manage..

[51] Messaoudi H, Gérard F, Dokukin P, Djamai H, Rebouh NY, Latati M (2020). Effects of intercropping on field-scale phosphorus acquisition processes in a calcareous soil. Plant Soil.

[52] Zheng Y, Liang J, Zhao DL, Meng C, Xu ZC, Xie ZH, Zhang CS (2020). The root nodule microbiome of cultivated and wild halophytic legumes showed similar diversity but distinct community structure in Yellow River Delta saline soils. Microorganisms.

[53] Matinzadeh Z, Akhani H, Abedi M, Palacio S (2019). The elemental composition of halophytes correlates with key morphological adaptations and taxonomic groups. Plant Physiol. Biochem..

[54] Mansouri M, Javadi SA, Jafari M, Arzani H (2021). Effect of microrelief and water-table on vegetation dynamics in silty loam saline soils of coastal areas. SN Appl. Sci..

[55] Schulz S, Darehshouri S, Hassanzadeh E, Tajrishy M, Schüth C (2020). Climate change or irrigated agriculture–what drives the water level decline of Lake Urmia. Sci. Rep..

